# Triptolide Decreases Cell Proliferation and Induces Cell Death in Triple Negative MDA-MB-231 Breast Cancer Cells

**DOI:** 10.3390/biom8040163

**Published:** 2018-12-05

**Authors:** Elizabeth Varghese, Samson Mathews Samuel, Sharon Varghese, Sohaila Cheema, Ravinder Mamtani, Dietrich Büsselberg

**Affiliations:** 1 Department of Physiology and Biophysics, Weill Cornell Medicine-Qatar, Education City, Qatar Foundation, P. O. Box 24144, Doha, Qatar; elv2007@qatar-med.cornell.edu (E.V.); sms2016@qatar-med.cornell.edu (S.M.S.); scv2002@qatar-med.cornell.edu (S.V.); 2 Institute for Population Health, Weill Cornell Medicine-Qatar, Education City, Qatar Foundation, P. O. Box 24144, Doha, Qatar; soc2005@qatar-med.cornell.edu (S.C.); ram2026@qatar-med.cornell.edu (R.M.)

**Keywords:** anticancer treatment, apoptosis, natural compounds, triple negative breast cancers, triptolide

## Abstract

Triple negative breast cancers (TNBCs) do not respond to conventional estrogen receptor/progesterone receptor/human epidermal growth factor receptor-2 targeted interventions due to the absence of the respective receptor targets. They are aggressive, exhibit early recurrence, metastasize, are more invasive in nature, and develop drug resistance. Some plant-derived substances have been screened and have gained attention as efficient anticancer drugs for TNBCs with few adverse effects. Here, we evaluate triptolide (concentrations in the range of 100 pM to 10 µM), a di-terpene tri-epoxide isolated from thunder god vine for its efficacy as anticancer drug in MDA-MB-231 TNBC cells. Cell proliferation and viability were assessed using 3-(4,5-dimethylthiazol-2-yl)-5-(3-carboxymethoxyphenyl)-2-(4-sulfophenyl)-2*H*-tetrazolium) (MTS) assay and trypan blue exclusion assay, respectively. A flow cytometry-based apoptosis assay was performed by using fluorescein isothiocyanate (FITC)-conjugated annexin V and propidium iodide (PI). Western blotting was performed to determine the levels of apoptotic and autophagy proteins such as caspase 3, LC3B and SQSTM1/p62. Results indicate that in 72 h of 1 nM triptolide treatment, the percentage of cell proliferation in MDA-MB-231 cells declined to 49 ± 18.9% (mean ± standard deviation (SD)), whereas the proliferation rate did not drop below 80% in MCF-7 cells (non-TNBC cells which express the estrogen receptor, progesterone receptor, and human epidermal growth factor receptor 2) even at the highest concentration tested (10 µM). The MDA-MB-468 cells showed a similar trend to MDA-MB-231 cells. Furthermore, triptolide treatment for 72 h significantly decreased cell viability at concentrations above 10 nM. Apoptotic cell death assay in 72 h triptolide-treated MDA-MB-231 cells revealed 29.3 ± 10.57% of early apoptotic cells in comparison to the control group (4.61 ± 2.24%). Cell cycle analysis indicated accumulation of cells in sub G_0_/G_1_ phase, indicating apoptosis. Western blot analysis in the 25 nM triptolide treatment group revealed induction of autophagy as shown by a significant decrease in the levels of autophagy marker p62 (by 0.2-fold *p* < 0.0001) and with an increase in the levels of LC3B-II (by 8-fold *p* < 0.05). An increase in the levels of the apoptotic marker cleaved caspase 3 (by 4-fold *p* < 0.05) was also observed in triptolide treated MDA-MB-231 cells. Our data shows that triptolide could be an efficient anticancer agent in the treatment of TNBCs.

## 1. Introduction

Breast cancer is the most common cancer, contributing to 15% of all cancer related deaths in women as estimated in 2018 (627,000 women died of breast cancer) [1]. Advances in diagnostics in recent years, especially in molecular biology and genomics of cancer, has revolutionized the detection of breast cancer [2]. New technology using microarrays has helped to identify prognostic and diagnostic markers and has identified several types and subtypes of breast cancer [3]. Molecular profiling of breast cancer broadly classifies breast cancer into hormonal receptor positive and negative breast cancer based on the presence or absence of three key bio-markers; the estrogen receptor (ER); the progesterone receptor (PR); and the human epidermal growth factor-2 (HER2) receptor [4]. Breast cancers devoid of these three key receptors are classified as triple negative breast cancers (TNBC) [5]. Although receptor targeting interventions for breast cancer treatment have shown positive outcomes for hormone receptor positive breast cancers, the lack of such specific targets in TNBCs leaves patients with only chemotherapy as the standard option for treatment [5]. Currently, the treatment regime for TNBC includes administration of anthracyclins, taxanes, and platinum drugs for early stage systemic treatment, and taxanes, topoisomerase II inhibitors and antimetabolites for metastatic cancer treatment [6,7,8,9,10]. More recently, the latest treatment strategy for TNBC includes immunotherapy, small molecule inhibitors such as poly adenosine diphosphate (ADP) ribose polymerase (PARP) inhibitors, mammalian target of rapamycin (mTOR) inhibitors, cyclin-dependent kinase 4 and 6 (CDK4/6) inhibitors, and androgen receptor-based hormonal therapy [10]. A recent review on chemotherapeutic agents used in metastatic breast cancer treatment gives an update on the classification of drugs and discusses the molecular targets in various signaling pathways, side effects, and resistance during the treatment [11,12]. A number of strategies for combating TNBC are underway with limited success and hence more efficient therapeutic regimes with minimal side effects are essential at this point. Fifteen to twenty percent of breast cancers are reported to be TNBCs [2]. Triple negative breast cancers are heterogeneous, very aggressive, and has poor prognosis [5]. Gene expression profiling has classified TNBC into six different molecular subtypes: (1) basal-like 1 (BL1), (2) basal-like 2 (BL2), (3) immunomodulatory (IM), (4) mesenchymal-like (M), (5) mesenchymal stem-like (MSL), and (6) luminal androgen receptors (LAR) subtypes [5]; which clinically vary in terms of selection and sensitivity to treatment, recurrence, and prognosis [5,13,14].

Plants are a fundamental source of phytochemicals which have been scientifically validated to have significant antiproliferative, antimetastatic, antiangiogenic, and pro-apoptotic effects in both animal and cell models [13,14]. A comprehensive review on selected natural compounds in TNBC treatment describes important signaling pathways in TNBC and potential targets of the compounds in different pathways [15,16,17]. A considerable amount of screening for potential anticancer agents has been undertaken and many successful anticancer drugs, such as paclitaxel, camptothecin, vinblastine, vincristine, and topotecan, have been isolated from plants [5]. Unlike the metal-based anticancer drugs, plant-derived compounds have shown reduced side effects and can enhance the efficacy in combination treatments and reverse drug-induced therapy resistance [5,18].

In this regard, triptolide, a di-terpene tri-epoxide isolated from the root extract of the Chinese herb, *Tripterygium wilfordii* of the Celastraceae family has gained importance as an efficient anticancer drug with minimal side effects [5]. Triptolide has been widely used in Chinese medicine for the treatment of rheumatoid arthritis, lupus, Behcet׳s disease, psoriasis, and central nervous system diseases [19]. Triptolide has a wide range of pharmacological properties, including antiproliferative and immunosuppressive properties, but its precise mechanistic action is not clearly understood. Scientific studies report the efficacy of triptolide in modulating multiple oncogenic and tumor suppressor pathways by targeting cellular targets such as cyclins, cyclin dependent kinases, caspases, heat-shock proteins, and proteins of the extracellular signal–regulated kinases (ERK), nuclear factor-kappa B (NF-κB), and angiogenesis pathways [20,21]. Triptolide treatment has been shown to be effective in the treatment of lung [22], prostate [23], gastric [24] pancreatic [25], and ovarian cancers [26], as well as leukemia [27]. Synergistic anti-cancer activity was observed when using a combination of triptolide and cisplatin which enhanced apoptosis in gastric cancer both in vitro and in vivo [28].

Triptolide treatment was associated with in vitro and in vivo cytotoxicity in human breast cancer stem cells and primary breast cancer cells [25]. The ERK activation-mediated induction of autophagy and apoptosis was reported in triptolide-treated Michigan Cancer Foundation-7 (MCF-7) breast cancer cells [29]. Triptolide-inhibited vascular endothelial growth factor (VEGF) induced angiogenesis in MDA-MB-231 and Hs578T breast cancer cells in vitro and decreased capillary density and cell proliferation in vivo in MDA-MB-231 cells injected into the mammary fat pad tumors of female nude mice [30]. Shaoet al. [31], reported Wnt/β-catenin signaling associated induction of apoptosis in triptolide treated MCF-7, BT-474, and MDA-MB-231 breast cancer cells. Another study reported an Akt inhibition-mediated anti-proliferative effect in triptolide-treated MDA-MB-468 cells [32]. Triptolide has also been shown to inhibit anti-apoptotic proteins X-linked inhibitor of apoptosis protein (XIAP) and cellular inhibitor of apoptosis protein1/2 (cIAP1/2). Scientific studies thus demonstrate multiple cell signaling pathways involved in triptolide treatment-associated antineoplastic effects in cancer cells.

In our current study, we have examined the effect of varying concentrations of triptolide on the proliferation of different breast cancer cell lines and we selected MDA-MB-231 (TNBC) cells for further investigating the mode of cell death by monitoring autophagy and apoptosis.

## 2. Materials and Methods

### 2.1. Cell Culture

The MDA-MB-231 (Cat. # HTB-26), MDA-MB-468 (Cat. # HTB-132), and MCF-7 (Cat. # HTB-22) breast cancer cells were purchased from the American Type Culture Collection (Manassas, VA, USA). Cells were grown in high-glucose Dulbecco’s modified eagle medium (DMEM) (Cat. # 11995; Thermo Fisher Scientific; Life Technologies Corporation, Grand Island, NY, USA) with 10% fetal bovine serum (FBS) (Cat # F2442; Merck/Sigma-Aldrich; St. Louis, MO, USA) and 1% penicillin–streptomycin (Cat. # 15140; Thermo-Fisher Scientific; Life Technologies Corporation, Grand Island, NY, USA).

### 2.2. Cell Proliferation Assay

The rate of cell proliferation was evaluated using CellTiter 96^®^ AQ_ueous_ One Solution Cell Proliferation Assay (Cat. # G3580; Promega, Madison, WI, USA). The reduction of the tetrazolium compound [3-(4,5-dimethylthiazol-2-yl)-5-(3-carboxymethoxyphenyl)-2-(4-sulfophenyl)-2H-tetrazolium, inner salt; MTS] by the dehydrogenase enzyme in the active cells yields a colored formazan compound which is read at 490 nM. The quantity of formazan product measured is directly proportional to the number of living cells in culture. The electron coupling reagent, phenazine ethosulfate (PES) present in the reagent enhances the chemical stability, allowing its combination with MTS to form a stable solution.

Briefly, cells for MTS assay were plated in 96-well plate at a concentration of 20,000 cells per well. The cells were incubated at 37 °C in a 5% CO_2_ incubator for 24 h, prior to triptolide treatment. Different concentrations of triptolide (100 pM to 10 µM) were applied and incubated for 24 h and 72 h time points. The cells were then incubated in 20 µL of CellTiter 96^®^ AQ_ueous_ One Solution reagent for another 30 min. The absorbance was read on a CLARIOstar spectrophotometer (BMG Labtech, Cary, NC, USA). The results were expressed as percentage of treated cells compared to untreated control using the equation: (% Viable = Absorbance_test_/Absorbance_control_ × 100). All the readings were normalized to the control and the control was considered 100% live cells. An average of five experiments was performed.

### 2.3. Trypan Blue Exclusion-Cell Viability Assay

Trypan blue dye (Cat. # 1450021; BioRad, Hercules, CA, USA) exclusion tests were carried out using a TC20 automated cell counter (Bio-Rad, Hercules, CA, USA). Phase contrast images of the cells were visualized using a 20× objective lens on Carl Zeiss epifluorescence microscope (Zeiss, Thornwood, NY, USA) and captured via a charge-coupled device (CCD) camera fitted to the microscope. The Zen Blue Lite software (Carl Zeiss Microscopy GmbH, Jena, Germany) was used to process the images.

### 2.4. Apoptosis Assay- Flow Cytometry

Annexin V–FITC and propidium iodide (PI) double staining technique was used to evaluate apoptosis using a FITC Annexin V Apoptosis Detection Kit (Cat. # 556547; BD Biosciences, San Jose, CA, USA). Annexin V binds to the exposed phosphatidylserine (PS) on the cell surface of apoptotic cells and PI stains the necrotic cells with the compromised cell membrane. For the assay, cells were seeded at a density of 0.5 × 10^6^ in a 100 mm dish. The cells after 72 h of triptolide treatment were trypsinized and washed in cold phosphate-buffered saline (PBS) and re-suspended in 100 µL annexin V binding buffer at a concentration of 1 × 10^6^ cells. To this 5 µL each of annexin V–FITC and PI were added. After gently vortexing, the cells were incubated for 15 min at room temperature (RT). Four hundred µL of annexin V binding buffer was added to the cells and the cells were analyzed on BD Fortessa flow cytometer (BD Biosciences, San Jose, CA, USA) using blue laser 488 nm. Data acquisition was performed using FACSDiva 6.3 software. FSC-Area (forward scatter) and SSC-Area (side scatter) gating was applied to discriminate single cell population from debris. The PMT for FSC-A and SSC-A were adjusted on untreated unstained cells. The PMTs for FITC and PI were defined using their respective single stained positive controls, staurosporin treated cells for apoptosis and heat-treated cells (56 °C for 3 min) for necrosis. The FITC and PI fluorescence were detected by 515 and 620 nm band filters, respectively. Automatic compensation was applied. Fifty-thousand events were recorded for each sample. The fluorescence was displayed on a scatter plot with PI and FITC quadrant gates. The population under each quadrant were exported to excel for further analysis. Data was averaged from at least five experiments.

### 2.5. Cell Cycle Analysis-Flow Cytometry

Cells were seeded in 100 mm dishes at a density of 0.5 × 10^6^ cells. After 72 h of treatment with triptolide, cells were trypsinized and centrifuged at 300× *g* for 5 min. The unattached cells were collected from the growth media and pooled. The cells were washed and re-suspended in PBS. The cells were fixed with absolute alcohol, replicates were accumulated, and stored at 4 °C in absolute alcohol until analysis [33]. On the day of analysis, the cells were centrifuged and re-suspended in propidium iodide (PI/RNase, Cat. # 4087S; Cell Signaling, Boston, MA, USA). Cells with the PI/RNase were incubated at 37 °C for 45 min. After incubation, cells were centrifuged, washed, and re-suspended in PBS. Data acquisition was carried out on BD Fortessa flow cytometry. Forward and side scatter area gating were used to identify singlets. Doublets were also discriminated by using forward scatter area and forward scatter height. Interval gates were placed on the detected peaks representing the phases of cell cycle on a PI histogram plot. Percentage of cells were estimated from each gate representing sub-G_0_/G_1_, G_0_/G_1_, S, G_2_/M phases of cell cycle.

### 2.6. Sodium Dodecyl Sulfate Polyacrylamide Gel Electrophoresis and Immunoblotting

Primary antibodies for SQSTM1/p62 (Cat. # 5114), LC3B (Cat. # 3868), cleaved caspase 3 (Cat. # 9664), pro-caspase 3 (Cat. # 9665), and β-actin (Cat. # 3700) were purchased from Cell Signaling Technology (Beverly, MSA, USA). The levels of SQSTM1/p62, LC3B, cleaved caspase 3 and pro-caspase 3 were analyzed by immunoblotting as described previously [33]. Briefly, cells for Western blotting were harvested from the 100 mm dish after 72 h of triptolide treatment. All steps of extraction process were performed at 2–8 °C. Both the attached and the floating cells were included in the extract. Cell lysate were extracted 1× radio-immunoprecipitation assay (RIPA) lysis buffer containing 150 mM NaCl, 1.0% IGEPAL^®^ CA-630, 0.5% sodium deoxycholate, 0.1% sodium dodecyl sulfate (SDS), 50 mM Tris, pH 8.0 (Cat. # R0278; Merck/Sigma-Aldrich, St. Louis, MO, USA) containing 10 μL/mL of Halt-protease and phosphatase inhibitors (Cat. # 78440; Thermo Fisher Scientific-Life Technologies Corporation) and 10 μL/mL 0.5 M ethylenediaminetetraacetic acid (EDTA), pH 8.0 (Cat. # 15575020; Thermo Fisher Scientific; Life Technologies Corporation, Waltham, MA, USA). The cells were centrifuged at 12,000× rpm at 4 °C for 10 min. The supernatant was separated and total protein was estimated using Bio-Rad DC protein assay kit (Cat # 500-0116, BioRad, Hercules, CA, USA). The required amounts of samples were mixed with 6× Laemmli SDS-reducing sample buffer containing bromophenol blue (Boston BioProducts, Ashland, MA, USA) and boiled for 5 min to ensure complete denaturation of the proteins. Twenty to twenty-five µg of protein were loaded on SDS gel (12%) and electrophoresed for 45 min at 200 V. The separated proteins were transferred to nitrocellulose membrane at 0.45 µM (Cat. # 1620115; BioRad) by the wet transfer method carried out at 100 V for 2 h. The membranes were blocked with 5% bovine serum albumin (BSA) in 1× tris-buffered saline (TBS)-Tween buffer for 1 h at room temperature. Subsequently, the blots were incubated with primary antibody over night at 4 °C on a rocker. On the following day, the blots were washed thrice in 1× TBS-Tween wash buffer and incubated in HRP conjugated secondary antibody for 1 h on a rocker at room temperature. The blots were then washed thrice in 1× TBS-Tween wash buffer and was developed using the enhanced chemiluminescent reagent and was developed using the Geliance 600 gel documentation system (PerkinElmer, Inc. Waltham, MA, USA). The band densities were analyzed using software Quantity One (basic) software (BioRad). β-actin was used as the loading control in all cases. The data shown is from four to five independent experiments.

### 2.7. Statistical Analysis

All data were analyzed with the statistical software GraphPad Prism 7.0 (GraphPad Software, Inc., CA, USA). Data are presented as mean ± standard deviation (SD). Statistical analysis was performed using Student’s *t*-test, one-way analysis of variance (ANOVA), and two-way ANOVA. Post-hoc comparisons between groups after ANOVA were performed by Tukey’s or Dunnett’s multiple comparison tests. *p*-values less than 0.05 (*p* < 0.05) were considered to be statistically significant.

## 3. Results

### 3.1. Time and Concentration Dependent Effect of Triptolide on Cell Proliferation in MDA-MB-231 Cells

**Figure 1:**
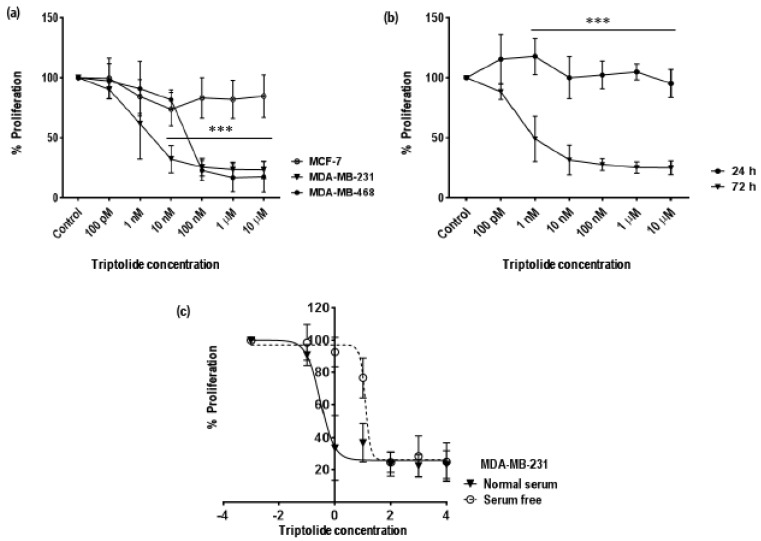
Effect of triptolide on cell proliferation in MDA-MB-231 cells. The line graph illustrates the effect of varying concentration of triptolide (100 pM to 10 µM) on cell proliferation (**a**) in MDA-MB-231, MDA-MB-468, and MCF-7 breast cancer cells after 72 h of triptolide treatment, (**b**) in MDA-MB-231 TNBC cells after 24 h and 72 h of treatment, and (**c**) in MDA-MB-231 cells after 72 h in treatment in the absence/presence of serum in the medium. Triptolide significantly reduced the cell proliferation in MDA-MB-231 cells when compared to MCF-7 cells, indicating that triptolide showed varied growth inhibitory effects in different breast cancer cell lines and the effect on MDA-MB-231 was more pronounced after 72 h of treatment. The presence of serum in the growth media enhanced the growth inhibitory action of triptolide in MDA-MB-231 cells, while serum withdrawal decreased the growth inhibitory effect. The inhibition of cell proliferation was significant between different groups with significance of ***p < 0.0001. MTS assay was used to evaluate the anti-proliferative effect of triptolide. Each graph represents pooled data from at least five experiments. The values are represented as mean ± standard deviation (SD).

Cell proliferation (MTS based) assay was performed to access anti-proliferative effect/cytotoxic effect of triptolide on hormone receptor positive and negative breast cancer cell lines. MCF-7 (ER, PR, HER2 positive) and TNBC cell lines MDA-MB-231 and MDA-MB-468 were treated with triptolide at concentrations of 100 pM, 1 nM, 10 nM, 100 nM, 1 µM, and 10 µM for 72 h. After treatment, the cell lines showed distinct differences in sensitivity to triptolide treatment. MDA-MB-231 showed more susceptibility to triptolide exposure when compared to the MCF-7 cells. Percentage change in rate of proliferation declined to 49 ± 18.9% (mean ± SD) and it further dropped below 25% for concentrations above 10 nM (*p* < 0.0001). In comparison to MDA-MB-231, MCF-7 was more stable to the treatment and triptolide was less cytotoxic (rate of proliferation 84 ± 17.61%), even at 10 µM, the highest concentration (Figure 1a).

Further analysis of MDA-MB-231 showed a time-dependent response and no significant response was recorded in the first 24 h of triptolide treatment (Figure 1b). However, a response was recorded after 72 h treatment. Next, we analyzed the effect of serum on the sensitivity of MDA-MB-231 cells to triptolide. MDA-MB-231 showed higher sensitivity to triptolide in the presence of serum than without serum in the medium (Figure 1c). Absence of serum from the media showed a distinct trend in the reduction of formazan. The half maximal inhibitory concentration (IC_50_) of triptolide in MDA-MB-231 under normal serum conditions was 0.3 nM and in serum free condition the IC_50_ increased to 12.7 nM. Nonlinear regression analysis was used to fit concentration–response data (Figure 1c) with log (inhibitor) vs. response–variable slope (four parameters).

### 3.2. MDA-MB-231 Showed Altered Morphological Characteristics Following Triptolide Treatment

**Figure 2:**
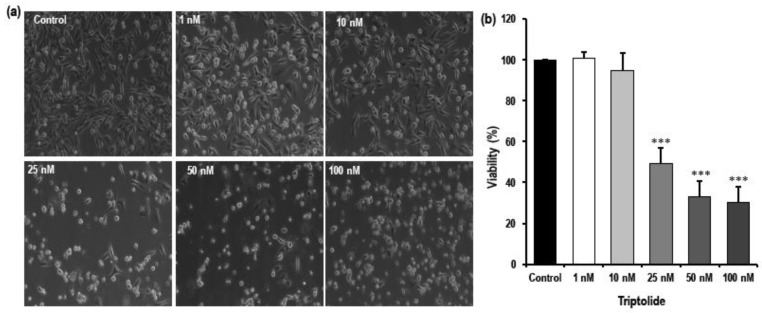
Cellular morphology and viability of MDA-MB-231 cells in response to triptolide treatment. (**a**) Representative phase contrast images of MDA-MB-231 under different concentrations of triptolide after 72 h of treatment. (**b**) Bar graph shows cell viability of MDA-MB-231 treated with varying concentrations of triptolide after 72 h of exposure. Morphological changes with the increasing concentrations of triptolide were observed after 72 h of treatment. The values are normalized to the control. Significant reduction in viability was observed in concentrations above 10 nM. The graph represents the average of at least five experiments.

The general morphology of the cells before and after treatment was studied using phase contrast microscopy (Figure 2a). The MDA-MB-231 under control conditions showed epithelial-like morphology with spindle shape and normal spreading. Severe changes in cell shape, detachment from the plate, and decrease in the number of cells were observed in MDA-MB-231 cells treated with triptolide for 72 h when compared to the non-treated control. To get more data points between the highest and the lowest dose response, 25 nM and 50 nM triptolide concentrations were added later in the study.

Cells showed indications of toxicity in concentrations from 25 nM to 100 nM. The concentration-dependent effect of triptolide on the cell viability was assessed using trypan blue. Figure 2b shows that the viability of the cells decreased significantly with increasing concentrations of triptolide. Lower concentrations, such as 1 nM and 10 nM, did not differ very much from the control. However, the viability decreased severely in concentrations above 10 nM. A 50% reduction in viability was observed in the 25 nM triptolide concentration (*p* < 0.0001). The viability was reduced by a further 30% in the 50 nM and 100 nM concentrations (*p* < 0.0001).

### 3.3. Triptolide Induces Apoptosis in MDA-MB-231 Cells

**Figure 3:**
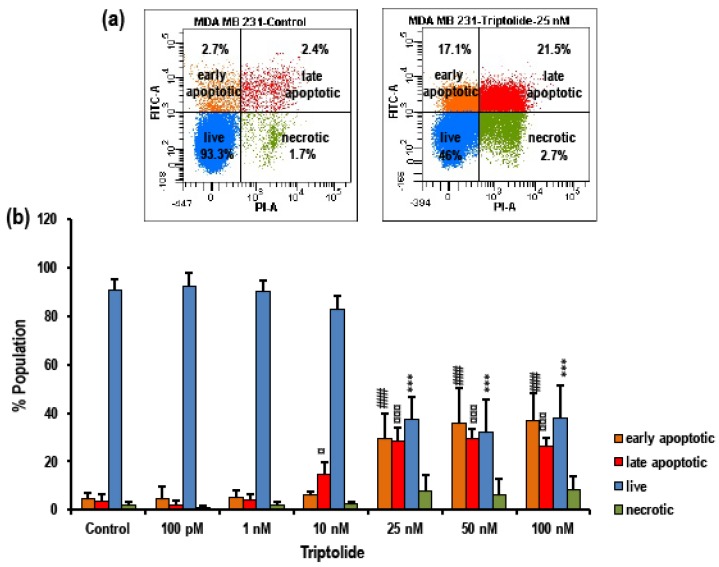
Effect of triptolide on cell death/apoptosis in MDA-MB-231 cells. (**a**) Representative scatter plots illustrating the live, early and late apoptotic, and necrotic cells in the untreated control and 25 nM triptolide-treated cells. (**b**) Bar graph shows consolidated data of live, early and late apoptotic, and necrotic population of control vs treated group. There is a significant increase in the apoptotic population in concentrations above 10 nM and the live population proportionally decreased. All experiments were repeated five times. The values are represented as mean ± SD. *** indicates *p* < 0.0001 for the live population; ### indicates *p* < 0.0001 for the early apoptotic population; # indicates *p* < 0.05 and ### indicated a *p* < 0.0001. All groups are compared to their corresponding controls.

Flow cytometry using annexin V–FITC and PI fluorescence staining was used to determine apoptosis (Figure 3a,b). The rate of apoptosis was significantly increased after treatment with triptolide for 72 h in comparison to the control group in MDA-MB-231.

In the control group, 91 ± 4.13% (mean ± SD) cells were live or viable cells, which were displayed as a FITC/PI-negative population in the scatter plot. The early apoptotic population which were FITC positive and PI negative comprised 4.61 ± 2.24% and the late apoptotic population which were both FITC- and PI-positive comprised 3.69 ± 2.58%. A 1.95 ± 1.17% proportion of the population was negative for FITC and positive for PI and were considered necrotic (Figure 3b). There was no sign of apoptosis after 72 h of triptolide treatment in the 100 pM and 1 nM concentration groups. The 10 nM treatment group showed a decrease in viability by 90 ± 5.46%, but this was not significantly different from the control group. However, the viability sharply dropped in the groups treated with 25 nM triptolide and above, followed by significant increase (*p* > 0.0001) in the apoptotic population, such as (29.3 ± 10.57%) early apoptotic, and 28.17 ± 5.77% late apoptotic (Figure 3b). There was no significant increase in the necrotic population in any of the treatment groups. An inverse trend between the viable population and the apoptotic population was observed in this analysis.

### 3.4. Triptolide Induces Accumulation of Cells in Sub-G_0_/G_1_ Phase Indicating Apoptosis in MDA-MB-231 Cells

**Figure 4:**
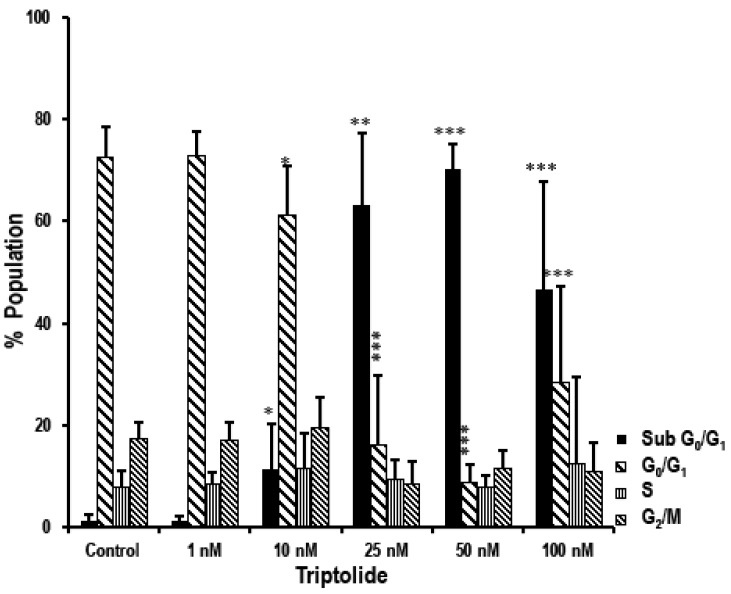
The effect of varying concentrations of triptolide on cell cycle. Bar diagram presents the cell cycle analysis of MDA-MB-231 cells. The population of cells, based on the florescent peaks, were grouped under sub G_0_/G_1_, G_0_/G_1_, S, G_2_/M phase. Untreated cells were used as controls. The MDA-MB-231 cells after 72 h of treatment with triptolide showed marked increase in the sub G_0_/G_1_ phase (in 10 nM and above), indicating an increase in cell death/apoptosis, and the proportion of cells in the G_0_/G_1_ phase decreased in comparison to the control. The values are represented as mean ± SD. *, *p*-value < 0.05; **, *p*-value < 0.001; ***, *p*-value < 0.0001.

Cell cycle phase distribution was analyzed by flow cytometry using PI staining to determine the cellular DNA content (Figure 4). A persistent high proportion of cells in sub-G_0_/G_1_ was observed in concentrations 25 nM and above, indicating apoptosis.

The S phase and G_2_/M phase did not show significant change under any treatment condition. However, in 10 nM (61.30 ± 9.41 vs. 72.56 ± 5.90; *p* < 0.05), 25 nM (16.17 ± 13.69 vs. 72.56 ± 5.90; *p* < 0.0001), 50 nM (8.83 ± 3.36 vs. 72.56 ± 5.90; *p* < 0.0001), and 100 nM (28.50 vs. 72.56 ± 5.90; *p* < 0.0001) triptolide-treated cells, there was a significant decrease in the percentage of cells in the G_0_/G_1_ phase when compared to the non-treated control.

### 3.5. Triptolide Induces Autophagy and Activates Apoptosis in MDA-MB-231 Cells

**Figure 5:**
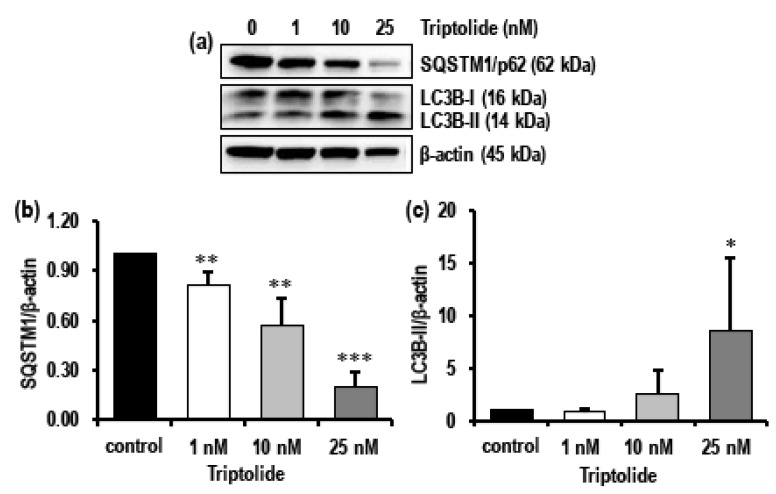
Effect of triptolide on the levels of SQSTM1/p62 and LC3B (autophagy related protein) in MDA-MB-231 cells after 72 h of triptolide treatment. (**a**) Representative Western blots show the levels of SQSTM1/p62 and LC3B in MDA-MB-231 cells treated with varied concentrations (1 nM, 10 nM, and 25 nM) of triptolide for 72 h. (**b**) Activation of autophagy was indicated by concentration-dependent decrease in level of SQSTM1/p62 and (**c**) an increase in the level of LC3B II. β-Actin was used as loading control. Values are expressed as mean ± SD from four to five experiments. Density was normalized to β-actin and ratio was calculated against untreated control. *, *p*-value < 0.05; **, *p* value < 0.001; ***, *p* value < 0.0001.

Western blot analysis revealed that 10 nM and 25 nM triptolide markedly activated autophagy in MDA-MB-231 cells as evidenced by the significant decrease in the levels of SQSTM1/p62, with 25 nM having the lowest expression (*p* < 0.0001), and an eight-fold increase in the levels of LC3B-II (*p* < 0.05) (Figure 5a–c).

**Figure 6:**
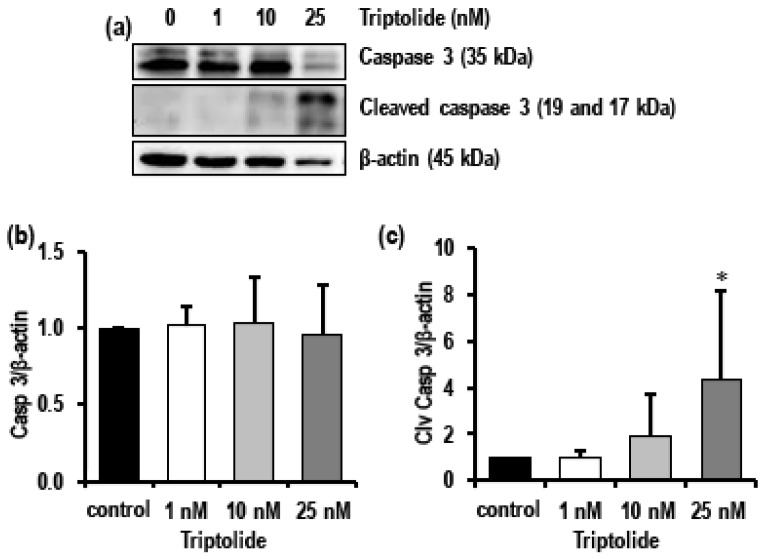
Effect of triptolide on the levels of caspase 3 and cleaved caspase in MDA-MB-231 cells after 72 h of triptolide treatment. (**a**) Representative Western blots show the levels of total caspase 3 and cleaved caspase 3 in MDA-MB-231 cells treated with varied concentrations (1, 10, and 25 nM) of triptolide for 72 h. (**b**,**c**) Bar graphs indicate the levels of caspase 3 and cleaved caspase 3 in triptolide-treated MDA-MB-231 cells. β-actin was used as loading control. Values are expressed as mean ± SD from four to five experiments. An increase in the level of cleaved caspase 3 in the treatment group indicated activation of apoptosis. Density was normalized to β-actin and the ratio was calculated against untreated control.

Initiation of apoptosis is marked by activation of caspase 3, followed by the increase in the level of cleaved caspase 3. In addition to triptolide-associated activation of dysregulated autophagy also activated apoptotic cell death as evidenced by increasing trend in the levels of cleaved caspase 3, while pro-/total caspase 3 levels decreased at 25 nM triptolide (Figure 6a–c).

## 4. Discussion

Triptolide is a bioactive compound isolated from *Tripterigium wilfordii*. Chemically, it is a di-terpenoid tri-epoxide. Triptolide has been reported to induce cell death in many types of cancers, such as lung, liver, breast, prostate, and ovarian cancers [33]. It induces apoptosis by modulation of multiple signaling pathways [24,34,35] via downregulation of pro-survival pathways and up regulation of different pro-apoptotic pathways.

Proliferation data from the MTS assay indicated time-, concentration-, and cell type-dependent effects of triptolide. The growth inhibitory effect of triptolide was more pronounced after 72 h treatment. The cytotoxicity of the drug in MDA-MB-231 cells was very high, even for concentration in the nM range, indicating a very low IC_50_ value of 0.3 nM in comparison to MCF-7 which showed 84.64% survival even at 10 µM, the highest concentration tested. Similar results were reported with 70% cell viability in 100 nM triptolide treatment after 48 h [36]. Conversely, a study reported 25% proliferation in MCF-7 cells after treatment with 400 nM/L triptolide [30]. Studies have reported that that the IC_50_ value of triptolide was 100-fold lower than anti-cancer drugs, such as doxorubicin and gemcitabine, widely used in breast and ovarian cancer treatment [19]. In our triptolide cell death assays, MTS assay data showed a slightly higher growth inhibitory effect (32% proliferation in 10 nM triptolide) when compared to cell death assays performed by trypan blue and fluorescence-activated cell sorting (FACS) analysis for apoptosis (94.8% proliferation and 82.8% live population in 10 nM triptolide). Talorete et al. [37], reported that type of flavonoids, growth medium, and serum components can influence the reduction of 3-(4,5-dimethylthiazol-2-yl)-2,5-diphenyltetrazolium bromide (MTT). Another group reported significant differences in MTT or MTS dyes for quantifying ATP and DNA, while testing the antiproliferative effect of EGCG ((-)epigallocatechin-3-gallate) in prostate cancer cells and MCF-7 breast cancer cells [38]. Hence it is important to report MTS/MTT assay results carefully, while taking other growth conditions into consideration. The effect of serum in the MTS assay was very marked in triptolide-treated MDA-MB-231 cells. In comparison to the serum free group, the presence of serum (10%) in the growth media enhanced the growth inhibitory effect by 40% in 1 nM and 10 nM triptolide concentrations. Study using annexin V–FITC and PI for apoptosis showed triptolide-induced cell death via apoptosis as indicated by a proportional increase in annexin V with increasing triptolide concentrations. However, there was no marked change in the rate of necrosis revealed by our FACS data. Our cell death assays show an inverse relationship between proliferation and apoptosis. The microscopic examination of triptolide-treated MDA-MB-231 cells showed indications of cell cytotoxicity which was comparable to our trypan blue and FACS apoptosis data. Cell cycle phase analysis of triptolide treated MDA-MB-231 cells showed accumulation of cells in sub-G_0_/G_1_ phase which correlated with our FACS apoptosis assay and reduction in the number of cells in G_0_/G_1_. Many reports indicate a G_0_/G_1_ arrest in triptolide-treated cancer cells [39]. In the current study, we have observed the cytotoxic effects of triptolide in MDA-MB-231 cells following 72 h of treatment with an increase in the number of cells in the sub-G_0_/G_1_ phase, indicating a halt in the cell cycle process.

Autophagy is a physiological process that controls cell turnover, and a number of scientific papers have reported autophagy as a protective mechanism in breast cancer cells [40,41,42]. Autophagy was confirmed in triptolide-treated MDA-MB-231 cells by a change in the levels of LC3B-II and p62. Though autophagy is reported as a protective mechanism in TNBC cancers, in our study, activation of autophagy following triptolide treatment was concomitantly correlated with the decrease in viability. Western blot analysis of LC3B, p62, caspase 3, and cleaved caspase 3 protein expression was modulated following triptolide treatment. Analysis of caspase 3, an effector caspase in the apoptotic cell death pathway, showed triptolide concentration-dependent activation in the increased expression of cleaved caspase 3. Western blot analysis for total caspase 3 and cleaved caspase 3 in triptolide-treated MDA-MB-231 cells confirmed activation of the apoptotic pathway and similar results supporting a caspase 3-dependent apoptotic mechanism has been reported in human gastric cancer BGC-823 cells and MCF-7 breast cancer cells [43,44]. In our study, increasing the dose of triptolide activated autophagy and induced apoptosis, leading to decreased MDA-MB-231 cell viability. However, adverse reactions related to hepatotoxicity and nephrotoxicity of triptolide have been reported in in vivo studies performed in mice [45]. Li et al. [46] reported that intraperitoneal injections of 1 mg/mL triptolide in BALB/C mice induced hepatocellular degeneration, increased alanine transaminase (ALT), aspartate transaminase (AST), and lactate dehydrogenase levels, indicating abnormal liver function. It was also reported that an activation of Nrf2 (transcription factor regulating the expression of antioxidant genes) could alleviate triptolide-induced hepatotoxicity. Safety and efficacy of triptolide as a potential anticancer drug should be carefully evaluated when considering clinical trials, since the effects of triptolide observed in both in vitro and in vivo (animal models) may not be necessarily translated completely in humans, since the mechanism of cancer progression and immune response in the clinical scenario involves more complex mechanisms.

## 5. Conclusions

Our study demonstrates that triptolide treatment in MDA-MB-231 cells activates the caspase 3-dependent apoptotic pathway and modulates autophagy signaling pathways, thus promoting cell death. Therefore, cross-talk between apoptosis and autophagy might be an important step in triptolide-mediated cell death in MDA-MB-231 cells. Aberrant autophagy, a protective mechanism in many cancers, can be re-programmed as a pro-apoptotic event with the intervention of appropriate chemotherapeutic agents. Further cell-based and in vivo studies are warranted in order to assess the effect of triptolide on multiple pathways that promote TNBC cell proliferation and inhibit cell death/apoptosis.
